# Taxonomic changes in the gut microbiota are associated with cartilage damage independent of adiposity, high fat diet, and joint injury

**DOI:** 10.1038/s41598-021-94125-4

**Published:** 2021-07-15

**Authors:** Kelsey H. Collins, Drew J. Schwartz, Kristin L. Lenz, Charles A. Harris, Farshid Guilak

**Affiliations:** 1 Department of Orthopaedic Surgery, Washington University, Couch Building Room 3213, 4523 Clayton Avenue, St Louis, MO 63110 USA; 2 Shriners Hospitals for Children, St. Louis, MO USA; 3 Center of Regenerative Medicine, Washington University, St. Louis, MO USA; 4 Division of Infectious Diseases, Department of Pediatrics, Washington University School of Medicine, St. Louis, MO USA; 5 Edison Family Center for Genome Sciences and Systems Biology, Washington University School of Medicine, St. Louis, MO USA; 6 Division of Endocrinology, Washington University, St. Louis, MO USA; 7 Present Address: Early Clinical Development & Experimental Sciences, Regeneron Pharmaceuticals, Tarrytown, NY USA

**Keywords:** Physiology, Musculoskeletal system, Osteoarthritis

## Abstract

Lipodystrophic mice are protected from cartilage damage following joint injury. This protection can be reversed by the implantation of a small adipose tissue graft. The purpose of this study was to evaluate the relationship between the gut microbiota and knee cartilage damage while controlling for adiposity, high fat diet, and joint injury using lipodystrophic (LD) mice. LD and littermate control (WT) mice were fed a high fat diet, chow diet, or were rescued with fat implantation, then challenged with destabilization of the medial meniscus surgery to induce osteoarthritis (OA). 16S rRNA sequencing was conducted on feces. MaAslin2 was used to determine associations between taxonomic relative abundance and OA severity. While serum LPS levels between groups were similar, synovial fluid LPS levels were increased in both limbs of HFD WT mice compared to all groups, except for fat transplanted animals. The Bacteroidetes:Firmicutes ratio of the gut microbiota was significantly reduced in HFD and OA-rescued animals when compared to chow. Nine novel significant associations were found between gut microbiota taxa and OA severity. These findings suggest the presence of causal relationships the gut microbiome and cartilage health, independent of diet or adiposity, providing potential therapeutic targets through manipulation of the microbiome.

## Introduction

The gut microbiome has been implicated in the pathogenesis of both rheumatoid arthritis and osteoarthritis (OA)^1^. Specifically, taxonomic changes in gut microbiota composition have been associated with OA onset and progression, especially with obesity^2,3^, a primary risk factor for OA^1,3^. While reports on the relationships between gut microbiota and cartilage damage to date have been largely correlative, fecal transplant from metabolically compromised human donors accelerates OA in germ-free mice challenged with a knee injury, suggesting this gut-cartilage axis plays a role in knee joint homeostasis^4^. In clinical populations, an analysis of the Rotterdam cohort revealed a significant positive relationship between joint pain and abundance of *streptococcus* species (spp.) in the gut microbiome, a finding that was replicated in the Lifelines-DEEP study^5,6^. This association was thought to be driven by local inflammation within the knee joint. However, because OA patients often have multiple co-morbidities^7^, there is a need to determine the disease-relevant gut microbial changes that are specifically involved in the pathogenesis of cartilage damage, while controlling for other factors.

Obesity is associated with several factors that potentially contribute to OA, such as gut microbiota dysbiosis, host responses from adipose tissue, circulating inflammatory mediators, and diet. However, the relative contributions of these factors in OA pathogenesis have been difficult to separate. The gut microbiota and its metabolites are key regulators of systemic inflammation from adipose tissue and are involved in the onset and entrenchment of metabolic disturbance^8^. Therefore, interrogating relations between outcomes of interest in the absence of a host-response from adipose tissue provides a direct approach for investigating the role of gut microbiota in diseases processes. Using a mouse model of lipodystrophy (LD), we demonstrated that many factors previously associated with the obesity-induced OA (i.e., synovitis, subchondral bone changes, inflammation) are indeed separable, as LD mice are protected from cartilage damage, and high fat feeding does not reverse the cartilage protection phenotype or worsen the metabolic dysregulation of LD mice fed a control diet^9^. Furthermore, cartilage protection in LD mice can be reversed by the implantation of a small transplant of adipose tissue. In this respect, comparing the gut microbiota across these conditions provides the opportunity to assess alterations in the gut microbiome while controlling for adipose tissue host-responses, obesogenic diet, and surgical onset of OA.

In the present study, we evaluated a subset of our previously reported cohort^9^ using 16S rRNA sequencing of the fecal microbiota in an attempt to separate factors previously associated with OA and obesity (i.e., synovitis, bone changes, inflammation) to evaluate relations between the gut microbiota and cartilage damage. We hypothesized that taxonomic changes in the gut microbiota are associated with OA severity when controlling for the effects of an obesogenic diet and of the presence of adipose tissue using LD mice. To add mechanistic insight to the evaluation of relations between the gut microbiota, the reintroduction of cartilage damage, and the absence of adipose tissue, we interrogated circulating and local levels of endotoxin, or lipopolysaccharide (LPS) in these animals^10^. This experimental design allows for the identification of gut microbiota changes that are associated with the reversal of cartilage protection and the onset of cartilage damage in this context.

## Materials and methods

All experimental procedures were carried out in accordance with the guidelines prescribed by the Washington University in St. Louis School of Medicine Department of Comparative Medicine, in compliance with the ARRIVE guidelines, and were approved by the Institutional Animal Care and Use Committee at Washington University in St. Louis School of Medicine.

LD mice were created by crossing adiponectin-Cre mice with homozygous lox-stop-lox-ROSA-diphtheria toxin A (DTA) mice^11^. DTA/ + wild-type (WT) littermates were maintained as controls. Male and female mice were fed either a chow control diet (10% kcal) or high fat diet (HFD, 60% kcal) from weaning. Further methodological details of this study can be found in the original paper detailing the cartilage phenotype of these mice^9^. Briefly, mice of different genotypes were co-housed to control for cage effects in gut microbiota composition. A subgroup of LD mice received either a mouse embryonic fibroblast transplant (MEF-R) between 3–5 weeks of age that forms an adipose-like tissue, or wildtype fat transplant containing visceral and subcutaneous fat (WF-R) between 6–8 weeks of age (n = 5–10 animals/sex/group). At 16-weeks, mice underwent surgery for destabilization of the medial meniscus (DMM) on their left knee, and the right limb served as a non-surgical contralateral control^9^. Intact lower limbs, serum, synovial fluid and a single fecal pellet from the rectum were collected immediately after sacrifice at 28 weeks of age^9^. To evaluate cartilage damage severity, safranin-O/fast green stained sections were assessed using the Modified Mankin Score, which have been reported previously^9^. For the present study, serum and synovial fluid LPS concentration was measured using a Pierce LAL Chromogenic Endotoxin Quantitation Kit (Thermofisher Scientific).

Fecal samples were analyzed using 16S rRNA amplicon sequencing to evaluate the microbial ecology of each sample on the Illumina NovaSeq Platform (performed by Mr. DNA, Shallowwater, TX). Further details can be found in Supplementary Methods. Operational taxonomic units (97% OTUs) were assigned using QIIME2 (www.qiime2.org) and^12^ classified using BLASTn against the NCBI database. Species richness, or the total number of unique OTUs within each sample, and Shannon Diversity Index, a measure of microbiome richness and evenness, was calculated using standard methods^13^ in R version 1.2.1073 (R Foundation for Statistical Computing, Vienna, Austria).

### Statistical analysis

Microbial taxonomic analysis was also performed using R version 4.0.2 and R studio version 1.3.1073 (R Foundation for Statistical Computing, Vienna, Austria)^13^. Bray–Curtis distance between microbial composition was calculated and principal coordinate analysis was used to visualize the data in these matrices. Pairwise analysis of similarities (ANOSIM) was utilized to determine significant differences in microbial community β-diversity. MaAslin2 was used to determine multivariable associations via generalized linear regression between taxonomic relative abundance and cartilage damage, operationalized by Modified Mankin Scores, by controlling for fixed effects of diet (chow or HFD), genotype (LD or WT), surgery and OA and random effect of individual samples^5,14^ with AST transformation of microbial abundance. LPS concentrations were analyzed by repeated measures ANOVA (genotype, limb, diet) and Tukey or Sidak post hoc testing with Graphpad Prism version 9 (Graphpad Software, La Jolla, Ca). We did not observe any significant differences in LPS levels, OA Scores, or microbiota $\alpha$ or β-diversity by sex, and therefore we evaluated male and female animals together within each group. P-values were corrected for multiple comparisons using Benjamini-Hochberg (BH) correction, $\alpha$=0.05.

## Results

**Figure 1:**
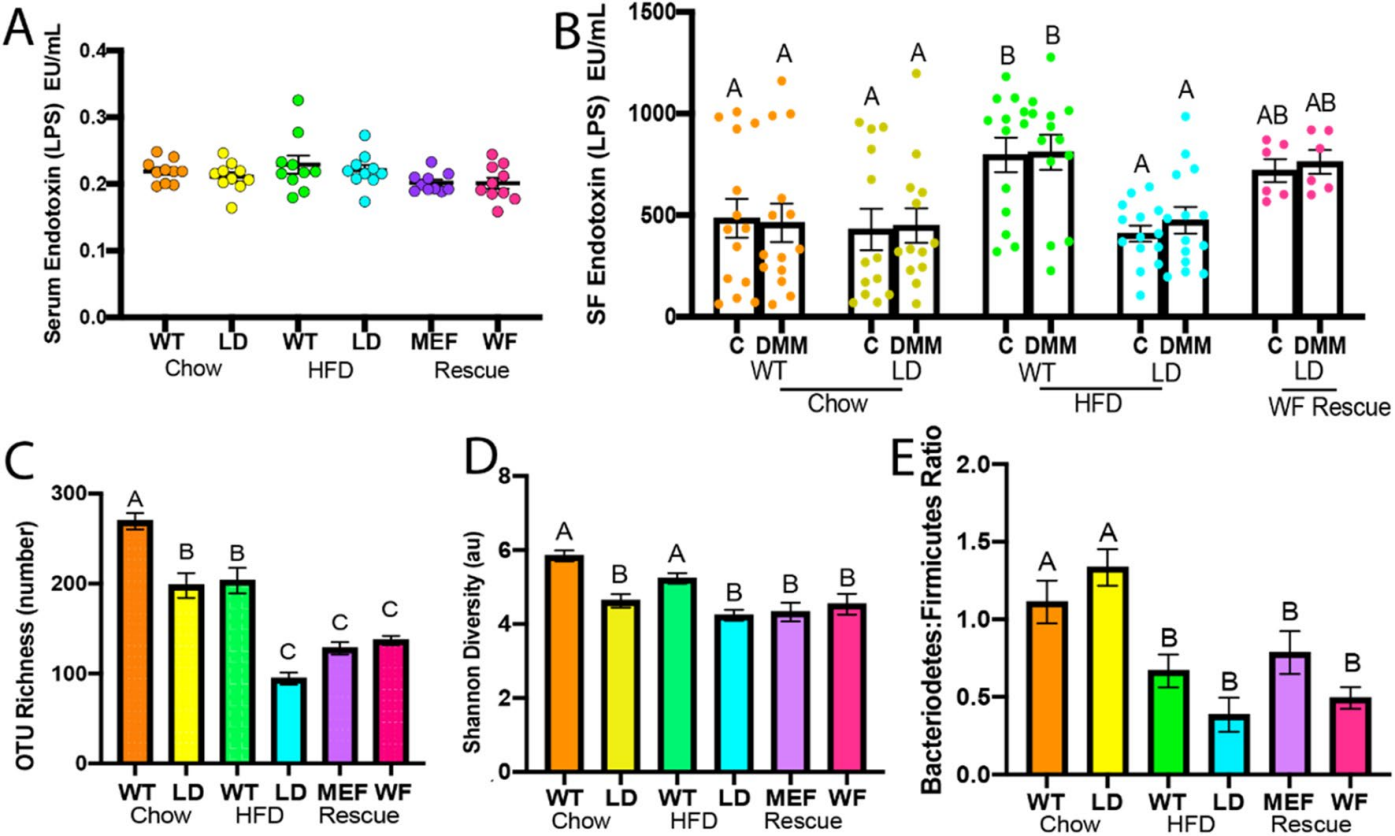
Chow-fed MEF and WF-rescued LD mice demonstrate increased diversity when compared to HFD-fed LD mice than WT or chow-fed LD mice, suggesting rescue does not recover taxonomic changes in gut microbiota. Serum levels for endotoxin, or lipopolysaccharide (**A**). Synovial fluid levels for lipopolysaccharide (**B**). Operational taxonomic units (OTU) richness (**C**) Shannon Diversity (**D**) and Bacteroidetes:Firmicutes Ratio (**E**) for all groups WT groups are displayed. Animal numbers by group: Chow WT n = 22, Chow LD n = 15, HFD WT n = 17, HFD LD n = 8, MEF-R n = 15, WF-R n = 12. Data were evaluated by one or two-way ANOVA and Tukey or Sidak post-hoc testing. Different letters indicate statistical significance, p < 0.05, such that A: p < 0.05 vs B and C; B: p < 0.05 vs A and C, C: p < 0.05 vs A and B; AB: p > 0.05 vs A and B.

There was no difference in serum LPS levels among groups (Fig. 1A). Synovial fluid levels for LPS were increased in both limbs of HFD-fed WT mice compared to all other groups, except for WF-R mice (Fig. 1B). However, WF-R mice LPS levels remained similar to all limbs from groups. Body composition, body mass, other synovial fluid, serum, and OA-related outcomes from these animals are reported elsewhere^9^.

OTU richness was decreased in all groups compared to chow-fed WT animals (Fig. 1C). Similarly, LD mice demonstrated decreased richness compared to HFD-fed WT animals. HFD-fed LD mice, MEF-R, and WF-R animals demonstrated further decreased richness compared to chow LD and HFD WT animals (p < 0.01). Shannon Diversity Index, a measure of evenness and richness, was similar between chow-fed LD, chow WT, and HFD WT animals, and decreased in LD, HFD LD, MEF-R, and WF-R animals (Fig. 1D). The ratio of Bacteroidetes to Firmicutes, a rough approximation of the ratio of anti-inflammatory to pro-inflammatory taxa, was similar in chow-fed LD and WT animals, but significantly reduced in HFD-fed WT, HFD-fed LD, WF-R, and MEF-R animals when compared to chow-fed WT (Fig. 1E).

**Figure 2:**
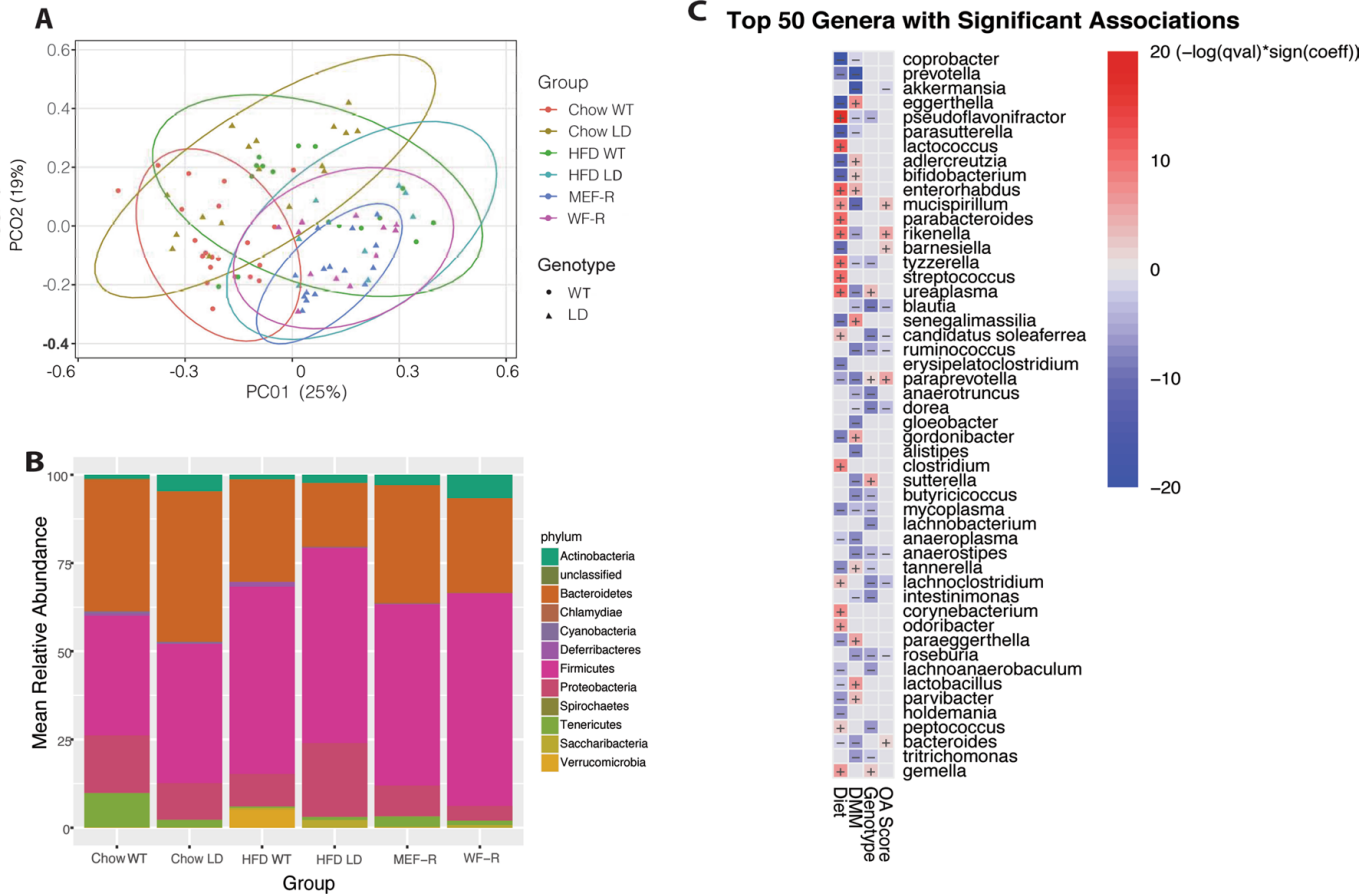
While distributions of MEF and WF-rescued LD mice are more similar to HFD-fed animals, nine genera were significantly associated with Modified Mankin Score. The first two PCoA vectors calculated by Bray–Curtis explained 44% of the variance in the groups (**A**), ellipses indicate 95% confidence intervals. Mean relative abundance at the phylum taxonomic level for each group (**B**). Heatmap of results from MaAslin2 indicating the magnitude and direction of significant associations between genera and fixed effects OA Score, Genotype, DMM, and HFD vs Chow Diet (**C**).

ANOSIM of the weighted UNIFRAC distance indicated significant differences between each treatment group with all other treatment groups (Supplementary Table 1, p < 0.05). Bray–Curtis dissimilarity matrices revealed that MEF-R and WF-R groups completely overlapped HFD LD suggesting more similar gut microbiome compositions (Fig. 2A). There was no overlap of 95% confidence intervals in this 2D space between MEF-R and chow-fed WT animals, and a minimal overlap between WF-R and chow-fed WT animals, suggesting that despite being fed a similar diet, MEF-R and WF-R did not restore taxonomic changes to that of chow-fed WT. Differences in the average relative abundance of several phyla between WT and LD mice were enhanced when animals were fed a HFD compared to chow (Fig. 2B). Specifically, Firmicutes were increased in both HFD and rescue groups (p < 0.01).

Multivariate generalized linear mixed effect models using MaAsLin2 indicated 9 significant associations of genera with cartilage damage determined by Modified Mankin Score (P < 0.05, BH correction, Table 1). The majority of these significant relationships with cartilage damage were negative, with *paraprevotella* spp*.* (Bacteroidetes phylum), *Citrobacter* spp. (Proteobacteria phylum), *dorea* spp. (Firmicutes phylum), *Anaerovorax* spp. (Firmicutes phylum), *Blautia* spp. (Firmicutes phylum), *Mucispirillum* (Deferribacteres phylum), and *Lachnoclostridium* spp. (Clostridium phylum*)*. Significant positive associations were observed with *Rikenella* spp. (Bacteroidetes phylum) and *Kingella* spp (Proteobacteria phylum). A much larger array of significant associations was found between the other fixed effects, specifically by diet (Supplementary Table 2). While *Streptococcus* spp. were not significantly associated with Modified Mankin Score, we did observe strong positive significant associations with high fat diet (Fig. 2C). Other significant relations by fixed effect, including genotype, DMM, and diet are summarized in a heatmap displayed in Fig. 2C.

**Table 1:** Summary of MaAslin2-derived significant associations between OTU abundance and cartilage damage, measured by Modified Mankin Score, when adjusted for diet, surgery, and genotype.

Species	Coefficient	Standard error	p-value	FDR p-value
*Paraprevotella* spp.	0.0129	0.0036	0.0007	0.0046
*Rikenella* spp.	0.0249	0.0071	0.0009	0.0052
*Kingella* spp.	0.0011	0.0003	0.0013	0.0071
*Citrobacter* spp.	-0.0055	0.0017	0.0017	0.0083
*Dorea* spp.	-0.0061	0.0021	0.0050	0.0205
*Anaerovorax* spp.	-0.0019	0.0007	0.0080	0.0303
*Blautia* spp.	-0.0052	0.0019	0.0082	0.0311
*Mucispirillum* spp.	0.0147	0.0055	0.0101	0.0357
*Lachnoclostridium* spp.	-0.0252	0.0100	0.0143	0.0481

P-value, and Standard Error from MaAsLin analysis.

*FDR* P-value adjusted for multiple testing, Benjamin–Hochberg false discovery rate.

## Discussion

We identified 9 novel taxonomic changes in gut microbiota composition that are associated with histological cartilage damage, when controlled for obesogenic diet and the presence of adipose tissue, revealing new targets for modulating gut microbiota-cartilage crosstalk involved in OA onset and progression. While MEF-R and WF-R restored susceptibility to cartilage damage in transplanted LD mice^9^, the gut microbiota from these chow-fed animals was more similar to HFD-fed WT and HFD-fed LD mice versus chow-fed LD mice. This is surprising as HFD is known to induce robust taxonomic microbiota alterations, and we previously found that MEF-R and WF-R partially reversed metabolic dysfunction, activity, muscle weakness, and alterations in bone microarchitecture in LD animals^9^.

Typically, species from the Bacteroidetes phylum are thought to exert largely anti-inflammatory actions, while firmicutes are thought to be largely proinflammatory^15^. As such, the ratio between these two phyla is commonly used as a proxy measure of the ratio between pro-inflammatory to anti-inflammatory microbes to illustrate anti-inflammatory shifts in taxonomic profiles from an insult or treatment. Here, we found that HFD-LD mice demonstrated a reduced Bacteroidetes:Firmicutes ratio compared to chow-fed groups, despite displaying protection from cartilage damage. These data suggest that this overall ratio may not be reflective of cartilage health, although typically an increased Bacteroidetes:Firmicutes ratio is associated with improved outcomes^2–5^. Moreover, negative associations with genera from both Bacteroidetes and Firmicutes phyla were observed to have significant negative associations with Modified Mankin Score, which demonstrates decreases in pro-inflammatory firmicutes spp. can co-occur with OA, and contributions to inflammation are perhaps time, context, and abundance-specific.

Endotoxemia has been hypothesized as a key host-response linking gut microbiota changes and OA^1,3,16^, and levels of LPS have been associated with OA severity in preclinical and clinical populations^3,10^. Systemically, gram-negative bacterially-derived LPS translocation can activate toll-like receptor 4 (TLR-4) in adipocytes and other host cells leading to systemic inflammation and metabolic disturbance, which may contribute to OA^3,16^. Furthermore, the gut microbiota may be influencing the joint more directly, or in the absence of a host responses through LPS or adipose tissue^1^. Within the joint, LPS is thought to prime pro-inflammatory macrophages through TLR-4 and is associated with activated synovial macrophages and knee OA severity^10^.

We did not observe differences in circulating levels of LPS between groups, suggesting that in this model system, systemic LPS is not associated with cartilage damage. However, we did observe local increases in LPS in the synovial fluid in HFD WT and partially in WF-R. Because WF-R LPS levels remained similar to that of chow-fed WT, chow-fed LD, HFD LD, and HFD-fed WT, and there were no increases in LPS in the DMM vs. non-surgical limbs, the relative contribution of SF LPS in the onset and progression of joint pathology is unclear. While it is possible that SF LPS plays an antagonistic role in joint damage we observed in HFD WT and the reversal of cartilage protection in WF-R, we would hypothesize that LPS levels would be increased in DMM limbs. More work is needed to make these determinations. Taxonomic changes we measured in this model may be more directly linked to cartilage damage, and perhaps not reliant on a host response (i.e. from adipose tissue). This also may provide partial support of the hypothesis that the gut bacteria-cartilage axis can be direct and not necessarily dependent on LPS signaling or host-responses in adipose^1^. However, the sample size for WF-R SF was restricted due to the secondary nature of this analysis, and SF samples available from MEF-R were not available, so our interpretation of the role of local LPS is this disease process is inherently limited. Furthermore, in the present analysis, we did not measure earlier timepoints where LPS levels could have fluctuated and influenced the cartilage damage that we ultimately measured at 28-weeks. Our future work will detail time course changes in synovial fluid of all groups considered here.

Several of the novel associations noted here are concordant with previous reports in the literature. *Anaerovorax* spp. are typically depleted in obese individuals, which is consistent with the significant negative relationship found here with OA when controlling for diet^17^. While the association between *Rikenella* spp. and cartilage damage is a novel association, *Kingella* spp. are common causes of septic arthritis and osteomyelitis in children^18^. *Rikenella* spp. and *Kingella* spp. are gram-negative species, so if they are indeed migrating to the joint through the circulation, could be contributing LPS within the joint directly^10^. *Blautia* spp. and *Dorea* spp. are known to produce butyrate, an anti-inflammatory metabolite that has been shown to modulate inflammation in chondrocytes through G Protein-coupled Receptor 43 (GPR43)^19^. While *Streptococcus* *spp.* were not found to be independently associated with OA when controlling for other factors as has been observed in other studies, we did observe a strong positive correlation with high fat diet^5^. It is possible that *Streptococcus* spp. may elicit a host response through adipose or other tissues that is not observed in the fat-free lipodystrophic mice, but future work is necessary to directly test this speculation.

Mitigating alterations in the composition of the gut microbiota using prebiotic fiber and exercise have been demonstrated to be protective from OA^2,20^, supporting the notion that modification of the gut microbiome could provide an attractive, minimally-invasive therapeutic target for cartilage damage^15,21,22^. For example, germ free mice demonstrate less bone loss in response to non-invasive load-based injury compared to conventional controls^23^. When germ free mice are challenged with DMM, the same injury used in the present study, they demonstrate significant reductions in osteophyte size and improvements in cartilage damage compared to conventional control mice, illustrating a role for the gut microbiota in promoting OA^24^. Furthermore, antibiotic treatment prior to injury has been shown to mitigate OA caused by a loading-induced injury^25^, as well as in toll-like receptor 5 knockout mice that are protected from metabolic syndrome^26^. Antibiotic-induced dysbiosis from treatment with ampicillin and neomycin reduced serum levels of LPS and improved OA outcomes after injury^27^, further illustrating that ablation of certain microbes could be a viable preventative treatment strategy for cartilage damage. Associations between the gut microbiome, muscle quality^28^, and bone strength^29^, contributors to OA pathogenesis^30^, have also been identified, suggesting that modulating the microbiome could have broad protective applications on the joint organ system. The present study contributes new knowledge to the identification of potential targets within gut microbiota for modification. It is possible that prebiotic fiber, probiotic supplementation, or repletion and/or selective depletion of the microbial taxa reported here could override cartilage protection LD mice, and future studies aim to determine cause-effect relationships in this context^1,15,31,32^.

This study has several potential limitations. By design, this study is a cross-sectional secondary analysis, and as such, we cannot deduce specific taxonomic changes involved throughout the progression of cartilage damage. Our synovial fluid analysis is limited in sample number and lacks the MEF-R group, so we cannot completely evaluate the potential increase in LPS and its role in the reversal of cartilage damage in fat rescued mice. Future efforts will chronicle time-course changes in fecal microbiota, synovial fluid, and serum after injury in LD, WT, and fat-rescued mice to understand taxonomic and endotoxin changes as they may occur with worsening joint damage. Furthermore, the LD mouse model is a well-controlled model system lacking adipose tissue, but as such, the insights identified here are context-dependent and need to be validated in other preclinical model systems. Finally, our use of 16s sequencing limits the interpretation of the present dataset to taxonomic profiling. Future work will employ shotgun metagenomic sequencing to interrogate metabolic function profiling.

In conclusion, this novel murine model of lipodystrophy can be used to separate the role of diet, adipose tissue host-response, and systemic inflammation, allowing analysis of links between gut microbiota and cartilage damage. We identified 9 novel associations between gut microbiota species and Modified Mankin Scores when controlling for inflammation and diet. The significant positive associations include: *Rikenella* spp. (Bacteroidetes phylum) and *Kingella* spp (Proteobacteria phylum). The significant negative associations include: *Paraprevotella* spp*.* (Bacteroidetes phylum), *Citrobacter* spp. (Proteobacteria phylum), *Dorea* spp. (Firmicutes phylum), *Anaerovorax* spp. (Firmicutes phylum), *Blautia* spp. (Firmicutes phylum), *Mucispirillum (*Deferribacteres phylum), and *Lachnoclostridium* spp. (Clostridium phylum*)*. This analysis identified targets that can be precisely modulated in future studies, colonizing germ free mice with specific microbial consortia, and/or selective addition or depletion of taxa within a community to understand the causal role between taxonomic alterations in the gut microbiome and cartilage health.

## Supplementary Information

Supplementary Information.

## Data Availability

Sequencing data has been deposited into the NCBI Sequence Read Archive (SRA) under Bioproject number PRJNA698353.
