# Supplementary material for: Taxonomic changes in the gut microbiota are associated with cartilage damage independent of adiposity, high fat diet, and joint injury
Source: Sci Rep. 2021 Jul 15;11:14560. doi: 10.1038/s41598-021-94125-4 (PMC8282619; doi:10.1038/s41598-021-94125-4)
Supplement: Supplementary file 1 — Supplementary Information. [file 41598_2021_94125_MOESM1_ESM.docx]

**Supplementary Information for**

Taxonomic Changes in the Gut Microbiota are Associated with Cartilage Damage Independent of Adiposity, High Fat Diet, and Joint Injury

Kelsey H. Collins, Drew J. Schwartz, Kristin L. Lenz, Charles A. Harris, Farshid Guilak

**Correspondence to:**

*Farshid Guilak, PhD

Email: guilak@wustl.edu

**This PDF file includes:**

Supplementary Tables S1-S2

Supplementary Methods

**Supplementary Table 1**. Pairwise ANOSIM of weighted UniFrac distance matrix with 999 permutations performed per between-groups comparison.

| Reference Group | Comparison Group | R | p-value | FDR p-value |
| --- | --- | --- | --- | --- |
| Chow WT | Chow LD | 0.38 | 0.001 | 0.001 |
| Chow WT | HFD WT | 0.57 | 0.001 | 0.001 |
| Chow WT | HFD LD | 0.81 | 0.001 | 0.001 |
| Chow WT | MEF-R | 0.37 | 0.001 | 0.001 |
| Chow WT | WF-R | 0.74 | 0.001 | 0.001 |
| Chow LD | HFD WT | 0.53 | 0.001 | 0.001 |
| Chow LD | HFD LD | 0.75 | 0.001 | 0.001 |
| Chow LD | MEF-R | 0.22 | 0.001 | 0.001 |
| Chow LD | WF-R | 0.28 | 0.003 | 0.003 |
| HFD WT | HFD LD | 0.34 | 0.001 | 0.001 |
| HFD WT | MEF-R | 0.42 | 0.001 | 0.001 |
| HFD WT | WF-R | 0.44 | 0.001 | 0.001 |
| HFD LD | MEF-R | 0.52 | 0.001 | 0.001 |
| HFD LD | WF-R | 0.53 | 0.002 | 0.002 |
| MEF-R | WF-R | 0.28 | 0.001 | 0.001 |

R scales from +1 to -1. A value of +1 indicating the most similar samples are in the same group. A value equal to 0 indicating there is no relationship observed between samples.

*Abbreviations:*

WT: Wildtype

LD: Lipodystrophic

HFD: High-fat diet

Chow: Chow diet

MEF-R: Mouse embryonic fibroblast rescued LD

WF-R: Wildtype fat rescued LD

FDR: P-value adjusted for multiple testing, Benjamin-Hochberg false discovery rate.

**Supplementary Table 2**. Summary of MaAslin2-derived significant associations between microbiota species and high fat diet, surgery, and genotype.

| **Species** | **Factor** | **Coefficient** | **Standard Error** | **P-value** | **FDR p-value** | **N** | **N>0** |
| --- | --- | --- | --- | --- | --- | --- | --- |
| *prevotella* spp. | surgery | -0.0883 | 0.0102 | 2.99e-12 | 6.81e-10 | 68 | 60 |
| *akkermansia* spp. | surgery | -0.0033 | 0.0004 | 1.14e-10 | 1.74e-08 | 68 | 68 |
| *mucispirillum* spp. | surgery | -0.0386 | 0.0066 | 2.02e-07 | 8.39e-06 | 68 | 26 |
| *ruminococcus* spp. | surgery | -0.0239 | 0.0049 | 7.47e-06 | 0.000162 | 68 | 67 |
| *paraprevotella* spp. | surgery | -0.0208 | 0.0043 | 9.95e-06 | 0.000189 | 68 | 68 |
| *gloeobacter* spp. | surgery | -0.0209 | 0.0044 | 1.34e-05 | 0.000236 | 68 | 68 |
| *alistipes* spp. | surgery | -0.0359 | 0.0077 | 1.78e-05 | 0.00029 | 68 | 68 |
| *sutterella* spp. | surgery | -0.0364 | 0.0080 | 2.53e-05 | 0.000361 | 68 | 67 |
| *butyricicoccus* spp. | surgery | -0.0039 | 0.0009 | 2.51e-05 | 0.000361 | 68 | 68 |
| *senegalimassilia* spp. | surgery | 0.0064 | 0.0014 | 3.09e-05 | 0.000414 | 68 | 66 |
| *eggerthella* spp. | surgery | 0.0078 | 0.0018 | 4.65e-05 | 0.000574 | 68 | 68 |
| *anaeroplasma* spp. | surgery | -0.0084 | 0.0019 | 5.52e-05 | 0.000662 | 68 | 68 |
| *anaerostipes* spp. | surgery | -0.0043 | 0.0010 | 6.75e-05 | 0.000789 | 68 | 68 |
| *ureaplasma* spp. | surgery | -0.0193 | 0.0045 | 7.38e-05 | 0.000842 | 68 | 68 |
| *roseburia* spp. | surgery | -0.0109 | 0.0026 | 0.000105 | 0.001022 | 68 | 59 |
| *lactobacillus* spp. | surgery | 0.1466 | 0.0356 | 0.000114 | 0.001063 | 68 | 65 |
| *bacteroides* spp. | surgery | -0.0734 | 0.0186 | 0.000206 | 0.001773 | 68 | 67 |
| *tritrichomonas* spp. | surgery | -0.0127 | 0.0032 | 0.000218 | 0.001843 | 68 | 66 |
| *kopriimonas* spp. | surgery | -0.0089 | 0.0023 | 0.000309 | 0.00242 | 68 | 57 |
| *helicobacter* spp. | surgery | -0.0714 | 0.0189 | 0.00035 | 0.002578 | 68 | 55 |
| *rikenella* spp. | surgery | -0.0318 | 0.0085 | 0.000393 | 0.002842 | 68 | 68 |
| *paraeggerthella* spp. | surgery | 0.0064 | 0.0018 | 0.000638 | 0.004219 | 68 | 34 |
| *gordonibacter* spp. | surgery | 0.0028 | 0.0008 | 0.00083 | 0.005114 | 68 | 68 |
| *parasporobacterium* spp. | surgery | -0.0106 | 0.0031 | 0.001043 | 0.006168 | 68 | 65 |
| *anaerotruncus* spp. | surgery | -0.0052 | 0.0016 | 0.001469 | 0.007564 | 68 | 68 |
| *enterorhabdus* spp. | surgery | 0.0068 | 0.0021 | 0.00173 | 0.008483 | 68 | 54 |
| *desulfomicrobium* spp. | surgery | -0.0144 | 0.0046 | 0.002688 | 0.012381 | 68 | 35 |
| *olsenella* spp. | surgery | 0.0410 | 0.0134 | 0.00318 | 0.014499 | 68 | 68 |
| *parvibacter* spp. | surgery | 0.0054 | 0.0018 | 0.004443 | 0.018989 | 68 | 68 |
| *subdoligranulum* spp. | surgery | 0.0015 | 0.0005 | 0.004494 | 0.018989 | 68 | 68 |
| *mycoplasma* spp. | surgery | -0.0479 | 0.0167 | 0.005586 | 0.022182 | 68 | 67 |
| *opitutus* spp. | surgery | -0.0037 | 0.0013 | 0.005594 | 0.022182 | 68 | 41 |
| *citrobacter* spp. | surgery | 0.0056 | 0.0020 | 0.006434 | 0.025075 | 68 | 68 |
| *adlercreutzia* spp. | surgery | 0.0067 | 0.0025 | 0.008459 | 0.031618 | 68 | 57 |
| *brachyspira* spp. | surgery | -0.0012 | 0.0004 | 0.008556 | 0.031722 | 68 | 10 |
| *pseudobutyrivibrio* spp. | surgery | -0.0185 | 0.0069 | 0.009914 | 0.035318 | 68 | 68 |
| *dehalobacterium* spp. | surgery | -0.0034 | 0.0013 | 0.011306 | 0.039356 | 68 | 67 |
| *blautia* spp. | surgery | -0.0059 | 0.0023 | 0.011757 | 0.040209 | 68 | 44 |
| *collinsella* spp. | surgery | 0.0015 | 0.0006 | 0.014332 | 0.048056 | 68 | 37 |
| *coprobacter* spp. | high-fat diet | -0.0191 | 0.0019 | 1.45e-14 | 6.59e-12 | 68 | 67 |
| *eggerthella* spp. | high-fat diet | -0.0234 | 0.0031 | 1.93e-10 | 2.20e-08 | 68 | 43 |
| *pseudoflavonifractor* spp. | high-fat diet | 0.0280 | 0.0037 | 2.67e-10 | 2.43e-08 | 68 | 67 |
| *parasutterella* spp. | high-fat diet | -0.1234 | 0.0186 | 9.08e-09 | 6.90e-07 | 68 | 30 |
| *lactococcus* spp. | high-fat diet | 0.1838 | 0.0295 | 4.27e-08 | 2.78e-06 | 68 | 64 |
| *adlercreutzia* spp. | high-fat diet | -0.0264 | 0.0043 | 5.82e-08 | 3.32e-06 | 68 | 45 |
| *bifidobacterium* spp. | high-fat diet | -0.1205 | 0.0201 | 1.15e-07 | 5.82e-06 | 68 | 68 |
| *enterorhabdus* spp. | high-fat diet | 0.0215 | 0.0036 | 1.35e-07 | 6.16e-06 | 68 | 67 |
| *parabacteroides* spp. | high-fat diet | 0.1083 | 0.0194 | 5.34e-07 | 1.87e-05 | 68 | 68 |
| *rikenella* spp. | high-fat diet | 0.0822 | 0.0147 | 5.29e-07 | 1.87e-05 | 68 | 35 |
| *barnesiella* spp. | high-fat diet | -0.1499 | 0.0273 | 7.38e-07 | 2.40e-05 | 68 | 68 |
| *tyzzerella* spp. | high-fat diet | 0.0237 | 0.0043 | 9.08e-07 | 2.61e-05 | 68 | 45 |
| *streptococcus* spp. | high-fat diet | 0.0302 | 0.0055 | 9.17e-07 | 2.61e-05 | 68 | 60 |
| *ureaplasma* spp. | high-fat diet | 0.0417 | 0.0079 | 1.62e-06 | 4.35e-05 | 68 | 51 |
| *senegalimassilia* spp. | high-fat diet | -0.0123 | 0.0025 | 4.43e-06 | 0.000106 | 68 | 68 |
| *erysipelatoclostridium* spp. | high-fat diet | -0.0048 | 0.0010 | 8.00e-06 | 0.000166 | 68 | 67 |
| *gordonibacter* spp. | high-fat diet | -0.0064 | 0.0014 | 1.69e-05 | 0.000286 | 68 | 41 |
| *prevotella* spp. | high-fat diet | -0.0820 | 0.0177 | 1.95e-05 | 0.000306 | 68 | 58 |
| *clostridium* spp. | high-fat diet | 0.1055 | 0.0229 | 2.06e-05 | 0.000313 | 68 | 14 |
| *mycoplasma* spp. | high-fat diet | -0.1306 | 0.0289 | 2.78e-05 | 0.000384 | 68 | 30 |
| *mucispirillum* spp. | high-fat diet | 0.0503 | 0.0114 | 4.31e-05 | 0.000545 | 68 | 68 |
| *tannerella* spp. | high-fat diet | -0.0945 | 0.0224 | 8.01e-05 | 0.000889 | 68 | 20 |
| *corynebacterium* spp. | high-fat diet | 0.0073 | 0.0017 | 8.57e-05 | 0.000889 | 68 | 68 |
| *odoribacter* spp. | high-fat diet | 0.0949 | 0.0228 | 9.65e-05 | 0.000957 | 68 | 65 |
| *paraeggerthella* | high-fat diet | -0.0128 | 0.0031 | 9.61e-05 | 0.000957 | 68 | 17 |
| *parvibacter* spp. | high-fat diet | -0.0129 | 0.0031 | 0.000118 | 0.001075 | 68 | 50 |
| *holdemania* spp. | high-fat diet | -0.0122 | 0.0030 | 0.000126 | 0.001128 | 68 | 51 |
| *gemella* spp. | high-fat diet | 0.0064 | 0.0016 | 0.000249 | 0.002066 | 68 | 14 |
| *ruminiclostridium* spp. | high-fat diet | -0.0148 | 0.0039 | 0.000323 | 0.002453 | 68 | 66 |
| *sporanaerobacter* spp. | high-fat diet | 0.0032 | 0.0008 | 0.000348 | 0.002578 | 68 | 68 |
| *catenibacterium* spp. | high-fat diet | -0.0424 | 0.0115 | 0.000476 | 0.003285 | 68 | 41 |
| *shigella* spp. | high-fat diet | 0.0359 | 0.0100 | 0.000665 | 0.00433 | 68 | 37 |
| *syntrophococcus* spp. | high-fat diet | -0.0061 | 0.0018 | 0.000868 | 0.005205 | 68 | 43 |
| *peptoclostridium* spp. | high-fat diet | -0.0228 | 0.0066 | 0.001055 | 0.006168 | 68 | 33 |
| *paraprevotella* spp. | high-fat diet | -0.0257 | 0.0075 | 0.001081 | 0.006238 | 68 | 9 |
| *acinetobacter* spp. | high-fat diet | 0.1016 | 0.0300 | 0.001219 | 0.006698 | 68 | 68 |
| *enterococcus* spp. | high-fat diet | 0.0093 | 0.0028 | 0.001239 | 0.006729 | 68 | 68 |
| *desulfovibrio* spp. | high-fat diet | 0.0771 | 0.0229 | 0.001286 | 0.006897 | 68 | 59 |
| *oscillospira* spp. | high-fat diet | 0.0412 | 0.0123 | 0.001396 | 0.007317 | 68 | 9 |
| *lachnoanaerobaculum* spp. | high-fat diet | -0.0029 | 0.0009 | 0.001476 | 0.007564 | 68 | 35 |
| *catabacter* spp. | high-fat diet | -0.0036 | 0.0011 | 0.002548 | 0.011914 | 68 | 59 |
| *oscillibacter* spp. | high-fat diet | 0.0083 | 0.0028 | 0.004256 | 0.018481 | 68 | 62 |
| *anaerovorax* spp. | high-fat diet | -0.0042 | 0.0014 | 0.004497 | 0.018989 | 68 | 43 |
| *candidatus.saccharimonas* spp. | high-fat diet | 0.0504 | 0.0172 | 0.004635 | 0.019392 | 68 | 30 |
| *coprococcus* spp. | high-fat diet | 0.0051 | 0.0017 | 0.004827 | 0.020009 | 68 | 66 |
| *desulfomicrobium* spp. | high-fat diet | 0.0232 | 0.0080 | 0.005035 | 0.020498 | 68 | 51 |
| *porphyromonas* spp. | high-fat diet | -0.0436 | 0.0157 | 0.007178 | 0.027504 | 68 | 68 |
| *papillibacter* spp. | high-fat diet | -0.0019 | 0.0007 | 0.008986 | 0.032674 | 68 | 9 |
| *anaerofustis* spp. | high-fat diet | -0.0019 | 0.0007 | 0.009028 | 0.032674 | 68 | 36 |
| *paludibacter* spp. | high-fat diet | -0.0066 | 0.0025 | 0.009554 | 0.034304 | 68 | 68 |
| *lachnoclostridium* spp. | high-fat diet | 0.0545 | 0.0206 | 0.010462 | 0.036698 | 68 | 23 |
| *candidatus.soleaferrea* spp. | high-fat diet | 0.0084 | 0.0032 | 0.011416 | 0.039437 | 68 | 33 |
| *staphylococcus* spp. | high-fat diet | 0.0627 | 0.0242 | 0.011816 | 0.040209 | 68 | 14 |
| *blautia* spp. | LD genotype | -0.0211 | 0.0041 | 3.14e-06 | 7.95e-05 | 68 | 68 |
| *candidatus.soleaferrea* spp. | LD genotype | -0.0168 | 0.0034 | 6.18e-06 | 0.000141 | 68 | 68 |
| *anaerotruncus* spp. | LD genotype | -0.0137 | 0.0028 | 9.57e-06 | 0.000189 | 68 | 51 |
| *dorea* spp. | LD genotype | -0.0217 | 0.0045 | 1.06e-05 | 0.000192 | 68 | 67 |
| *lachnobacterium* spp. | LD genotype | -0.0033 | 0.0007 | 3.28e-05 | 0.000428 | 68 | 33 |
| *lachnoclostridium* spp. | LD genotype | -0.0914 | 0.0218 | 8.58e-05 | 0.000889 | 68 | 68 |
| *intestinimonas* spp. | LD genotype | -0.0079 | 0.0019 | 8.55e-05 | 0.000889 | 68 | 68 |
| *lachnoanaerobaculum* spp. | LD genotype | -0.0039 | 0.0009 | 0.000109 | 0.001032 | 68 | 33 |
| *peptococcus* spp. | LD genotype | -0.0136 | 0.0034 | 0.000144 | 0.001262 | 68 | 34 |
| *anaerosporobacter* spp. | LD genotype | -0.0042 | 0.0011 | 0.000265 | 0.002156 | 68 | 68 |
| *oscillospira* spp. | LD genotype | -0.0496 | 0.0130 | 0.000308 | 0.00242 | 68 | 44 |
| *spirochaeta* spp. | LD genotype | -0.0080 | 0.0021 | 0.000313 | 0.00242 | 68 | 23 |
| *oscillibacter* spp. | LD genotype | -0.0109 | 0.0029 | 0.000438 | 0.003122 | 68 | 50 |
| *anaerovorax* spp. | LD genotype | -0.0056 | 0.0015 | 0.000471 | 0.003285 | 68 | 68 |
| *sutterella* spp. | LD genotype | 0.0534 | 0.0146 | 0.000517 | 0.00352 | 68 | 68 |
| *subdoligranulum* spp. | LD genotype | -0.0035 | 0.0010 | 0.000541 | 0.003629 | 68 | 17 |
| *dehalobacterium* spp. | LD genotype | -0.0084 | 0.0024 | 0.000805 | 0.005096 | 68 | 68 |
| *ruminococcus* spp. | LD genotype | -0.0314 | 0.0089 | 0.000824 | 0.005114 | 68 | 43 |
| *marvinbryantia* spp. | LD genotype | -0.0107 | 0.0031 | 0.00116 | 0.006613 | 68 | 23 |
| *roseburia* spp. | LD genotype | -0.0163 | 0.0048 | 0.001211 | 0.006698 | 68 | 20 |
| *citrobacter* spp. | LD genotype | -0.0124 | 0.0037 | 0.001191 | 0.006698 | 68 | 32 |
| *pseudobutyrivibrio* spp. | LD genotype | -0.0416 | 0.0127 | 0.00166 | 0.008319 | 68 | 22 |
| *parasporobacterium* spp. | LD genotype | -0.0185 | 0.0056 | 0.001648 | 0.008319 | 68 | 20 |
| *mycoplasma* spp. | LD genotype | -0.0985 | 0.0305 | 0.001936 | 0.00939 | 68 | 22 |
| *butyricicoccus* spp. | LD genotype | -0.0050 | 0.0016 | 0.002015 | 0.009674 | 68 | 20 |
| *anaerostipes* spp. | LD genotype | -0.0059 | 0.0018 | 0.002315 | 0.010998 | 68 | 68 |
| *hespellia* spp. | LD genotype | -0.0067 | 0.0021 | 0.00256 | 0.011914 | 68 | 67 |
| *pseudoflavonifractor* spp. | LD genotype | -0.0120 | 0.0039 | 0.003465 | 0.015643 | 68 | 24 |
| *tyzzerella* spp. | LD genotype | -0.0137 | 0.0046 | 0.003939 | 0.017609 | 68 | 68 |
| *candidatus.stoquefichus* spp. | LD genotype | -0.0022 | 0.0007 | 0.004043 | 0.017899 | 68 | 28 |
| *butyrivibrio* spp. | LD genotype | -0.0061 | 0.0021 | 0.004245 | 0.018481 | 68 | 10 |
| *robinsoniella* spp. | LD genotype | -0.0038 | 0.0013 | 0.005079 | 0.020498 | 68 | 68 |
| *fastidiosipila* spp. | LD genotype | -0.0030 | 0.0010 | 0.005881 | 0.023116 | 68 | 20 |
| *ureaplasma* spp. | LD genotype | 0.0233 | 0.0083 | 0.006559 | 0.025346 | 68 | 18 |
| *sporobacter* spp. | LD genotype | -0.0075 | 0.0028 | 0.008875 | 0.032636 | 68 | 45 |

P-value and Standard Error from MaAsLin analysis.

FDR: P-value adjusted for multiple testing, Benjamin-Hochberg false discovery rate.

**Supplemental Methods**

The 16S rRNA primer pair, 515F GTGYCAGCMGCCGCGGTAA/ 806R GGACTACNVGGGTWTCTAAT, was used to evaluate each sample on the NovaSeq with under the bTEFAP® DNA analysis service offered by Mr. DNA Stillwater, TX. PCR was conducted on each sample for 30 cycles using HotStarTaq Plus Master Mix Kit (Qiagen, Valencia, CA) under the following conditions: 95°C for 5 minutes, followed by 30 cycles of 95°C for 30 seconds; 53°C for 40 seconds and 72°C for 1 minute. A final elongation step at 72°C for 10 minutes was performed. All amplicon products from different samples were mixed in equal concentrations and purified using SPRI beads and sequenced with the Illumina NovaSeq in accordance with manufacturer’s protocols.

The Q25 sequence data was processed using an established analysis pipeline (www.mrdnalab.com, MR DNA, Shallowater, TX). Briefly, sequences are depleted of primers, small sequences < 150bp are removed, and sequences with ambiguous base calls removed. Sequences are quality filtered (maximum expected error threshold of 1.0) and dereplicated. The dereplicated or unique sequences are denoised, unique sequences identified with PCR point errors are removed, followed by chimera removal, thereby providing a denoised sequence or zOTU. Final zOTUs were taxonomically classified using BLASTn against the NCBI database (www.ncbi.nlm.nih.gov) and compiled into each taxonomic level to generate relative abundance.
